# Influence of OATP1B1 and BCRP polymorphisms on the pharmacokinetics and pharmacodynamics of rosuvastatin in elderly and young Korean subjects

**DOI:** 10.1038/s41598-019-55562-4

**Published:** 2019-12-19

**Authors:** Yun Kim, Seonghae Yoon, Yewon Choi, Seo Hyun Yoon, Joo-Youn Cho, In-Jin Jang, Kyung-Sang Yu, Jae-Yong Chung

**Affiliations:** 1 Department of Clinical Pharmacology and Therapeutics, Seoul National University College of Medicine and Hospital, Seoul, Korea; 2 Clinical Trials Center, Seoul National University College of Medicine and Bundang Hospital, Seongnam, Korea; 3 Clinical Pharmacology and Therapeutics, Seoul National University College of Medicine and Bundang Hospital, Seongnam, Korea

**Keywords:** Clinical pharmacology, Pharmacodynamics, Pharmacokinetics, Dyslipidaemias, Genetics research

## Abstract

A lack of information regarding whether genetic polymorphisms of *SLCO1B1* and *ABCG*2 affect the pharmacokinetics (PKs)/pharmacodynamics (PDs) of rosuvastatin in elderly subjects prevents optimal individualized pharmacotherapy of rosuvastatin in clinical settings. This study aimed to investigate the effect of age and genetic polymorphisms and possible differences in genetic effects on the PKs/PDs of rosuvastatin between elderly and young subjects. Two separate clinical studies designed as open-label, one-sequence studies with multiple-dose administration for elderly (n = 20) and young (n = 32) subjects were conducted. All subjects received 20 mg of rosuvastatin once daily for 21 days. The exposure to rosuvastatin, characterized by the area under the time curve (AUC), increased by 23% in the elderly subjects compared with that of young subjects, which was not significant. When compared to the subjects with breast cancer resistance protein (BCRP) normal function, the exposure to rosuvastatin increased by 44% in young subjects (p = 0.0021) with BCRP intermediate function (IF) and by 35% and 59% (p > 0.05 for both) in elderly subjects with BCRP IF and low function, respectively. *SLCO1B1* 521T > C was also partially associated with a higher AUC of rosuvastatin in young subjects and a less pronounced increasing trend in elderly subjects (p > 0.05 for both). The lipid-lowering effect of rosuvastatin was less pronounced in the elderly subjects than in the young subjects, and genetic polymorphisms of neither *SLCO1B1* nor *ABCG2* significantly affected the PDs of rosuvastatin. The *ABCG2* 421C > A polymorphism was associated with the PKs of rosuvastatin and was identified as a more important determinant than the *SLCO1B1* 521T > C polymorphism in both elderly and young subjects.

## Introduction

Rosuvastatin is one of the most hydrophilic statins (3-hydroxy-3-methyl-glutaryl (HMG)-CoA reductase inhibitors) used for the treatment of hyperlipidemia^1^. The elimination of rosuvastatin appears to occur primarily via the liver^2^; however, metabolism is not a major factor contributing to its elimination^3–6^, and multiple drug transporters are known to be involved in the hepatic uptake and efflux of rosuvastatin. Rosuvastatin is taken up into the liver predominantly via the organic anion-transporting polypeptide 1B1 (OATP1B1, gene *SLCO1B1*), which is expressed in the basolateral membrane of human hepatocytes and is involved in the uptake of diverse endogenous and foreign compounds into the hepatocyte^7,8^. Additionally, rosuvastatin is a substrate of the breast cancer resistance protein (BCRP, gene *ABCG2*), which is an efflux transporter expressed in various normal tissues, including the small intestine, colon, liver and kidney^9–11^. BCRP functions as a rate-determining barrier to the absorption of its substrates from the gut and increases the excretion of its substrates into the bile and urine, thereby potentially reducing systemic exposure to many drugs, including rosuvastatin^11,12^. Rosuvastatin also undergoes active renal elimination mediated by organic anion transporter 3, which is responsible for active tubular secretion^13^.

Drug transporters are considered to be important determinants of rosuvastatin pharmacokinetic/pharmacodynamic (PK/PD) profiles, and genetic polymorphisms of OATP1B1 and BCRP are known to be associated with interindividual variability in the PKs/PDs of rosuvastatin^14–16^. Through its effect on altered rosuvastatin absorption, the 421C > A single-nucleotide polymorphism (SNP) in *ABCG2* appears to be a major genetic factor associated with the PKs of rosuvastatin, with higher exposure compared with that in noncarriers of this allele^12^. Similarly, the T521 > C SNP of *SLCO1B1* shows a relationship with a higher plasma concentration of rosuvastatin compared with that in noncarriers of this allele.^14,17^

However, there is currently a lack of information regarding whether genetic polymorphisms of OATP1B1 and BCRP affect the PKs/PDs of rosuvastatin in elderly subjects. Martin *et al*. reported that differences of less than 15% in rosuvastatin PKs were identified between healthy elderly and young groups, which was not considered clinically relevant^18^. Furthermore, with regard to the PDs of rosuvastatin, similar percentage reductions in low-density lipoprotein cholesterol (LDL-c) have been observed in elderly and younger hypocholesterolemic patients^19–21^.

This study was conducted to determine whether genetic polymorphisms of OATP1B1 and BCRP influence PKs/PDs after multiple-dose administration of rosuvastatin in elderly subjects and to compare the results with those of young subjects.

## Results

### Study characteristics

A total of 32 young subjects completed the study. Of the two subjects who dropped out, one withdrew their consent before the initial dosing and the other withdrew consent due to a generalized urticaria as an adverse event. All elderly subjects completed the study. Mean lipid levels and body mass index (BMI) were relatively higher in the elderly subjects than in the young subjects (Table 1).

**Table 1:** Baseline characteristics of 34 young and 20 elderly subjects who completed the clinical trials according to OATP1B1 and BCRP phenotypes.

OATP1B1 BCRP	Young							Elderly						p-value
	NF		IF		LF		Total	NF			IF		Total	
	NF	IF	NF	IF	NF	IF		NF	IF	LF	NF	IF		
N	11	10	7	4	1	1	34	7	8	1	2	2	20	—
Age (year)	27.5 ± 5.1	26.3 ± 5.9	29.6 ± 6.1	23.8 ± 1.0	26.0	24.0	27.0 ± 5.2	70.7 ± 5.6	71.3 ± 2.8	68.0	69.5 ± 0.7	77.5 ± 2.1	71.4 ± 4.3	<0.001^a^
Sex (M/F)	10/1	8/2	6/1	3/1	1/0	0/1	28/6	7/0	8/0	1/0	2/0	2/0	20/0	0.074^b^
Height (cm)	174.7 ± 7.0	173.0 ± 6.2	170.4 ± 9.5	168.8 ± 7.0	169.8	162.8	172.1 ± 7.3	168.3 ± 4.7	162.1 ± 5.2	157.7	165.1 ± 7.9	160.3 ± 0.1	164.2 ± 5.6	<0.001^c^
Body weight (kg)	68.9 ± 9.1	66.5 ± 9.1	66.1 ± 14.3	62.1 ± 8.2	65.4	51.5	66.2 ± 10.1	71.7 ± 6.0	68.5 ± 6.0	61.8	64.8 ± 0.2	63.0 ± 8.1	68.4 ± 6.2	0.335^c^
BMI (kg/m2)	22.5 ± 1.7	22.1 ± 2.1	22.6 ± 3.0	21.8 ± 2.1	22.7	19.4	22.2 ± 2.1	25.3 ± 1.6	26.1 ± 1.4	24.9	23.9 ± 2.2	24.5 ± 3.1	25.4 ± 1.7	<0.001^c^
LDL-c (mg/dL)	108.8 ± 34.6	110.7 ± 27.7	121.9 ± 33.2	95.5 ± 16.5	127.0	92.0	110.5 ± 29.6	130.9 ± 36.1	111.1 ± 23.0	102.0	117.5 ± 2.1	113.5 ± 36.1	118.5 ± 27.8	0.335^c^
TC (mg/dL)	170.2 ± 32.0	170.2 ± 31.4	177.7 ± 25.3	144.8 ± 9.6	197.0	165.0	169.4 ± 28.6	211.0 ± 45.7	187.1 ± 26.5	171.0	197.0 ± 26.9	183.0 ± 35.4	195.3 ± 34.5	0.004^c^
TG (mg/dL)	91.2 ± 29.8	80.1 ± 28.4	121.3 ± 57.3	66.5 ± 12.2	103.0	54.0	90.5 ± 38.1	172.6 ± 146.8	110.9 ± 26.4	209.0	103.0 ± 22.6	156.0 ± 41.0	141.1 ± 91.4	0.008^a^
HDL-c (mg/dL)	53.5 ± 9.1	53.3 ± 14.5	47.0 ± 10.4	46.5 ± 5.1	62.0	69.0	52.0 ± 11.3	50.4 ± 12.8	51.4 ± 7.2	42.0	51.5 ± 10.6	43.0 ± 2.8	49.8 ± 9.3	0.460^c^

Values are presented as the means ± standard deviation or the number of subjects. Comparison between total young and elderly subjects was performed each by ^a^Mann-Whitney U test or ^b^Chi-square test or ^c^Two sample t-test.

NF, normal function; IF, intermediate function; LF, low function; N, number of subjects; BMI, body mass index; LDL-c, Low-density lipoprotein cholesterol; TC, Total cholesterol; TG, Triglycerides; HDL-c, High-density lipoprotein cholesterol.

### Genetic polymorphisms of OATP1B1 and BCRP

A total of 52 subjects (32 young and 20 elderly) gave consent for pharmacogenetic analysis of *SLCO1B1* and *ABCG2*. The polymorphism of each genotype of the subjects in this study was similar to that obtained from 442 Koreans^22^, except for *SLCO1B1* 388A > G due to all the young subjects being identified as G/G.

### PK analysis of rosuvastatin

After administration of 20 mg of rosuvastatin for 21 days, the maximum observed concentration at steady state (C_max,ss_) and the area under the concentration-time curve from 0 to the dosing interval of 24 h for rosuvastatin (AUC_tau,ss_) increased by 9.6% and 23%, respectively (p > 0.05), in the elderly subjects compared with the same parameters in the young subjects. The median time to maximum plasma concentration at steady state (T_max_) was relatively longer in the elderly subjects (5 h) than in the young subjects (4 h) in the total/total and normal function (NF)/NF groups (Table 2, p = 0.0265 and 0.0449, respectively), and no significant differences in T_max_ were identified in the other groups.

**Table 2:** Pharmacokinetic parameters of rosuvastatin in young and elderly subjects after oral administration of 20 mg of rosuvastatin for 21 days according to OATP1B1/BCRP phenotypes.

OATP1B1/BCRP phenotypes	T_max,ss_ (h)		C_max, ss_ (μg/L)		AUC_tau,ss_ (μg*h/L)		t_1/2_ (h)		CL_ss_/F (L/h)	
	Young	Elderly	Young	Elderly	Young	Elderly	Young	Elderly	Young	Elderly
**Total population**										
Total/Total (n = 32) vs (n = 20)	4.0 [2.0–5.0]	5.0 [2.0–5.0]	39.6 ± 14.9 (37.6)	43.4 ± 20.4 (47.0)	367 ± 126 (34.2)	450 ± 184 (40.9)	9.1 ± 2.5 (27.0)	8.0 ± 2.1 (26.8)	61.5 ± 22.8 (37.0)	52.7 ± 25.2 (47.8)
	0.0265		NS		NS		NS		NS	
**OATP1B1 NF**										
NF/NF (n = 10) vs (n = 7)	4.0 [2.0–5.0]	5.0 [3.0–5.0]	30.1 ± 7.9 (26.3)	37.2 ± 13.8 (37.2)	276 ± 70.1 (25.4)	388 ± 155 (40.0)	9.1 ± 1.6 (17.4)	8.9 ± 3.3 (37.1)	77.9 ± 24.5 (31.4)	61.8 ± 32.3 (52.3)
	0.0449		NS		NS		NS		NS	
NF/IF (n = 9) vs (n = 8)	5.0 [3.0–5.0]	5.0 [2.0–5.0]	42.7 ± 14.6 (34.2)	42.6 ± 8.0 (18.8)	428 ± 132 (30.8)	455 ± 107 (23.5)	10.2 ± 3.5 (33.8)	7.3 ± 0.8 (11.5)	50.9 ± 16.0 (31.3)	46.7 ± 13.2 (28.3)
	NS		NS		NS		NS		NS	
NF/LF (None) vs (n = 1)	—	5.0	—	49.0	—	595.0	—	8.3	—	33.6
**OATP1B1 IF**										
IF/NF (n = 7) vs (n = 2)	3.9 [2.0–4.0]	2.0 [2.0–2.0]	37.3 ± 14.3 (38.4)	37.1 ± 20.0 (54.0)	329 ± 105 (31.8)	321 ± 169 (52.8)	8.4 ± 2.9 (34.6)	5.9 ± 0.2 (3.7)	66.2 ± 20.5 (31.0)	72.5 ± 38.3 (52.8)
	NS		NS		NS		NS		NS	
IF/IF (n = 4) vs (n = 2)	4.5 [4.0–5.0]	4.0 [3.0–5.0]	45.9 ± 9.6 (20.8)	72.3 ± 62.2 (86.0)	434 ± 87.3 (20.1)	705 ± 425 (60.3)	8.4 ± 0.8 (9.7)	7.5 ± 1.8 (24.5)	47.6 ± 9.9 (20.8)	34.7 ± 20.9 (60.3)
	NS		NS		NS		NS		NS	
**OATP1B1 LF**										
LF/NF (n = 1) vs (None)	4.0	—	58.0	—	460	—	9.2	—	43.5	—
LF/IF (n = 1) vs (None)	3.0	—	79.1	—	621	—	7.8	—	32.2	—
OATP1B1 NF vs IF	NS		NS	0.0299	NS	NS	NS		NS	
BCRP NF vs IF			0.0295	NS	0.0021					
NF/NF vs NF/IF			0.0399		0.0096					
NF/NF vs IF/IF			0.0347		0.0259					

Data are expressed as mean values ± standard deviation (CV %) except for T_max,ss_ shown as the median [range].

C_max,ss_, maximum observed concentration at steady state; AUC_tau,ss_, area under the concentration-time curve from 0 to the dosing interval of 24 h for rosuvastatin; CL_ss_/F, oral clearance at steady state; t_1/2_, half-life; T_max,ss_, time to maximum plasma concentration at steady state; NF, normal function; IF, intermediate function; LF, low function; n, number of subjects; NS, not significant.

**Figure 1:**
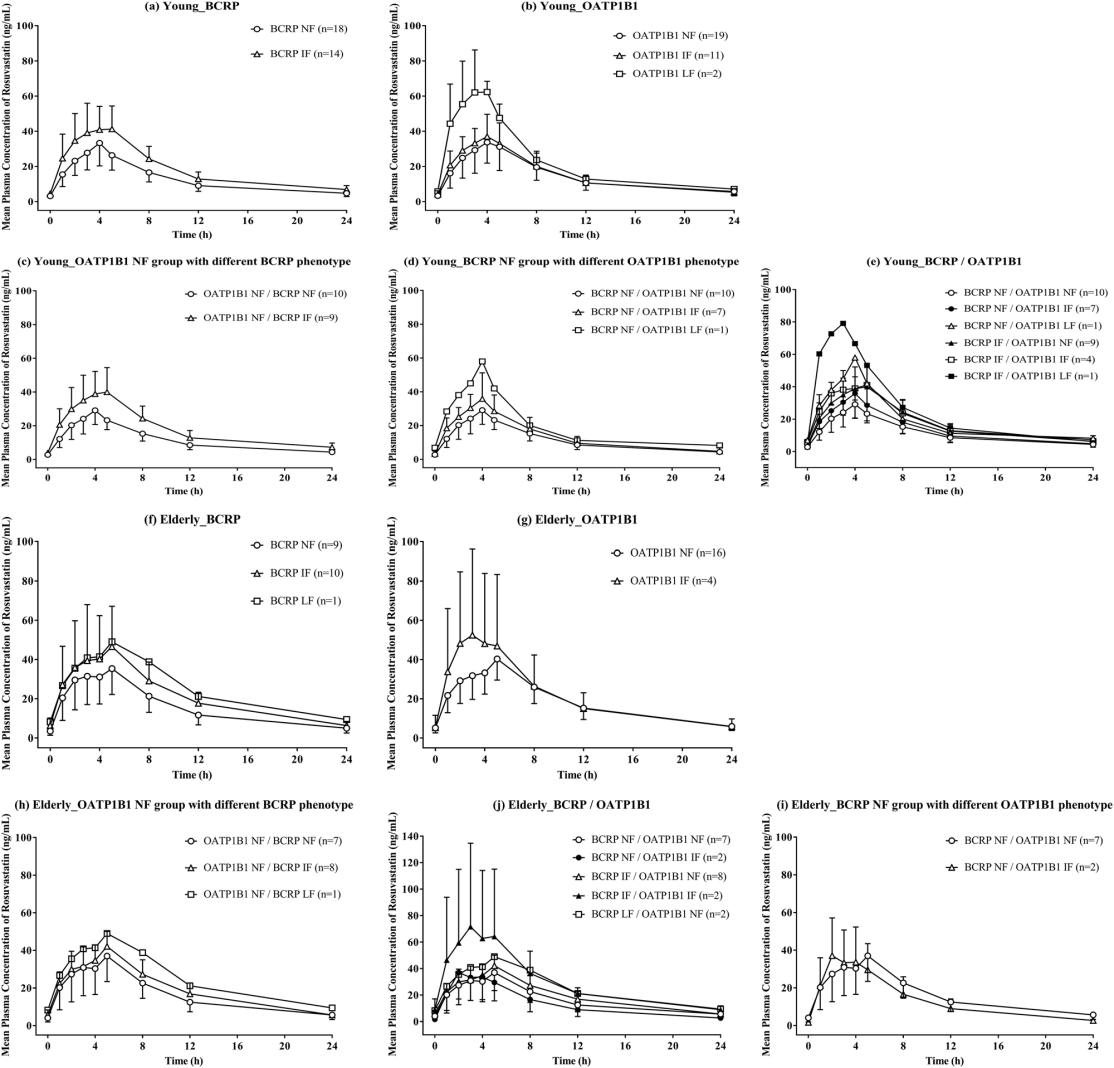
Mean plasma concentration–time profiles of rosuvastatin according to BCRP and OATP1B1 phenotype in young (n = 32) and elderly (n = 20) subjects. (**a,f**) BCRP; (**b,g**) OATP1B1; (**c,h**) OATP1B1 NF group with different BCRP phenotype; (**d,i**) BCRP NF group with different OATP1B1 phenotype; (**e,j**) BCRP/OATP1B1. Bars represent standard deviations.

**Figure 2:**
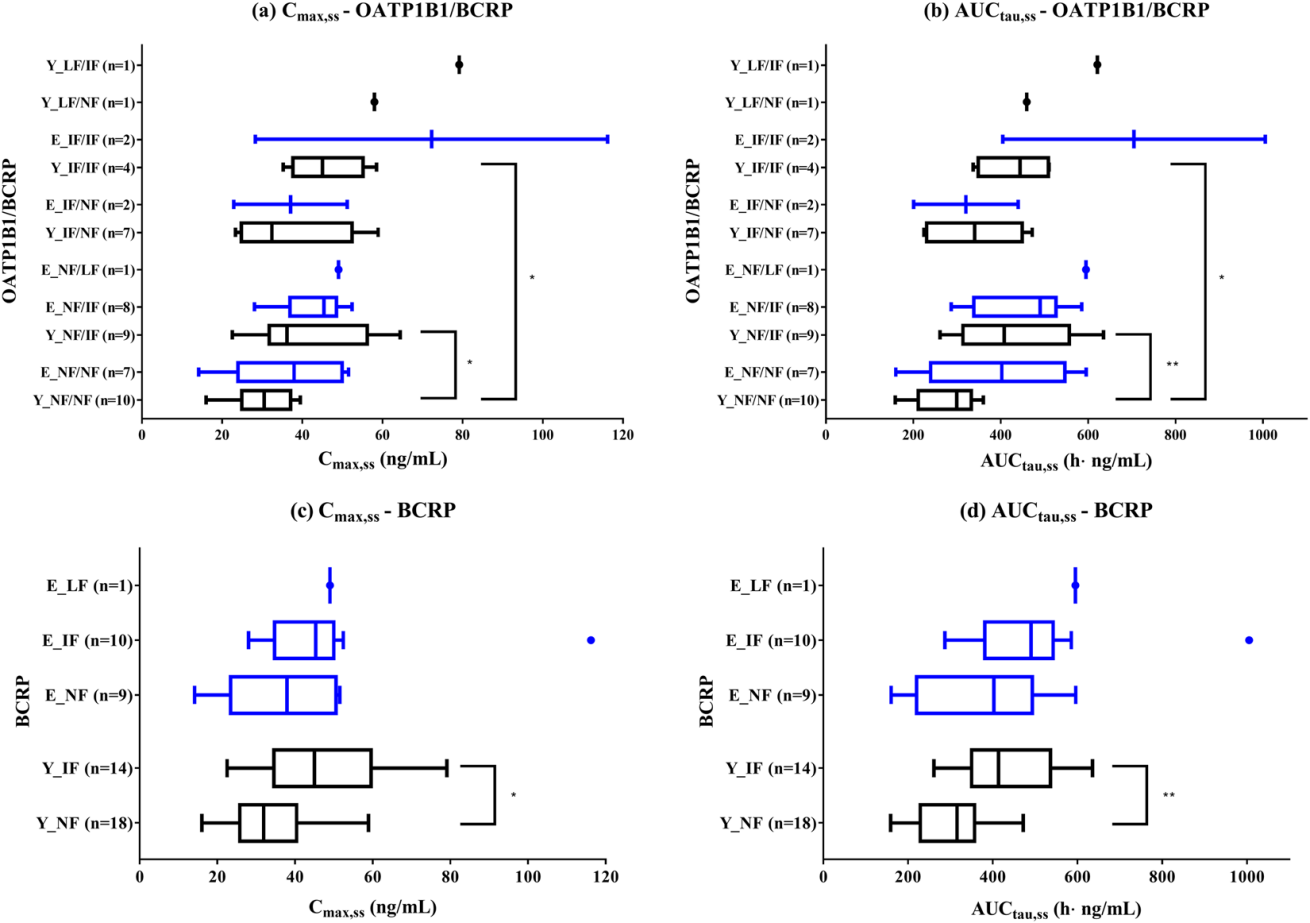
Box-and-whiskers plots of pharmacokinetic parameters (**a,c**) C_max,ss_; (**b,d**) AUC_tau,ss_ after oral administration of 20 mg rosuvastatin for 21 days according to OATP1B1/BCRP and BCRP phenotypes in young (Y) and elderly (E) subjects. The vertical lines within each box represent the median. The box edges show lower (25th) and upper (75th) quartiles, respectively. The whiskers and outliers were plotted using the Tukey method. *Represents p value < 0.05, **represents p value < 0.01.

In both elderly and young subjects, the mean plasma concentrations of rosuvastatin generally increased as the function of BCRP and OATP1B1 decreased [Fig. 1(a,b,f,g)]. When we only considered the reduced function of BCRP (OATP1B1/BCRP groups: NF/NF vs. NF/intermediate function (IF) vs. NF/low function (LF), IF/NF vs. IF/IF, and LF/NF vs. LF/IF), exposure to rosuvastatin increased in both the elderly and young subjects (Figs. 1, 2 and Table 2). For the young subjects, C_max,ss_ and AUC_tau,ss_ were significantly greater in the NF/IF (42%, p = 0.0399; 55%, p = 0.0096, respectively) and IF/IF groups (53%, p = 0.0347; 57%, p = 0.0259, respectively) than in the NF/NF group [Fig. 2(a,b)]. In the young subjects, C_max,ss_ and AUC_tau,ss_ were also significantly greater in the BCRP IF group (34%, p = 0.0295; 44%, p = 0.0021, respectively) than in the BCRP NF group [Fig. 2(c,d) and Table 2]. Similarly, C_max,ss_ and AUC_tau,ss_ in elderly subjects increased as the function of BCRP decreased [p > 0.05 for both BCRP IF (31% and 35%) and LF (32% and 59%)]. In both elderly and young subjects, the median T_max_ ranged from 4.0 to 5.0 h, even among those with different BCRP phenotypes (Table 2, NF/NF, NF/IF, and NF/LF).

Multiple linear regression analysis showed that only the reduced function of BCRP significantly contributed to a higher AUC_tau,ss_ of rosuvastatin in the final model (p = 0.0397, Supplementary Table S1). The finally selected model explained 6.4% of the variability. The other independent variables, including reduced function of OATP1B1, age (or class), sex, BMI, serum creatinine level, and concomitant medications or diseases, were not significantly associated with the AUC_tau,ss_ of rosuvastatin.

When we only considered the reduced function of OATP1B1 (OATP1B1/BCRP groups: NF/NF vs. IF/NF vs. LF/NF, NF/IF vs. IF/IF), the exposure to rosuvastatin remained similar in the elderly subjects, whereas exposure increased in the young subjects (p > 0.05 for both: NF/NF vs. IF/NF, 19%; NF/NF vs. LF/NF, 66%) [Fig. 2(a,b) and Table 2]. Notably, for T_max_, there was a reducing trend as the function of OATP1B1 decreased in both the young (p > 0.05) and the elderly subjects (median T_max_: 5.0 h for OATP1B1 NF, 2.5 h for IF; p = 0.0299). The effects of other genetic polymorphisms of *SLCO1B1* on PKs were estimated to be small but could not be confirmed conclusively (Supplementary Table S2).

### PD analysis of rosuvastatin

**Figure 3:**
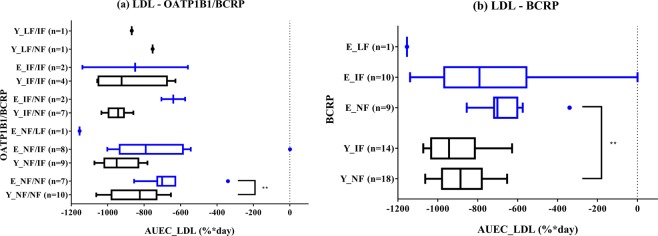
Box-and-whiskers plots of area under the effect-time curve (AUEC) of low-density lipid (LDL) after oral administration of 20 mg rosuvastatin for 21 days according to (**a**) OATP1B1/BCRP and (**b**) BCRP phenotypes in young (Y) and elderly (E) subjects. The horizontal lines within each box represent the median. The box edges show lower (25th) and upper (75th) quartiles, respectively. The whiskers and outliers were plotted using the Tukey method. **Represents p value < 0.01.

We observed a tendency whereby the lipid-lowering effect of rosuvastatin was less pronounced in the elderly subjects when compared to that in the young subjects (Fig. 3 and Table 3). No apparent relationship was observed between PD parameters [maximum change from baseline (%) and area under the effect-time curve (AUEC) (%·day) in lipids] and OATP1B1/BCRP phenotypes. For LDL-c, as a primary PD parameter of rosuvastatin, the elderly subjects showed lower absolute AUECs than the young subjects in the Total/Total, NF/NF, and BCRP NF groups (19%, p = 0.0107; 21%, p = 0.0063; and 25%, 0.0013, respectively). Furthermore, the elderly subjects showed a lower absolute maximum change in LDL-c from baseline than the young subjects in the NF/NF group (11%, p = 0.0330). The absolute AUECs of LDL-c tended to be greater as the function of BCRP decreased [Fig. 3(b)] but was not statistically significant in either young or elderly subjects. Except for the IF/IF group, absolute maximum changes in triglycerides (TGs) from baseline in the elderly subjects tended to be lower than those in the young subjects, and the most marked change was observed in the NF/IF group (young: -38% vs. elderly: -28%, p = 0.0464). Interestingly, the absolute AUECs of high-density lipoprotein cholesterol (HDL-c) in the elderly subjects were significantly lower than those in the young subjects in the Total/Total and NF/IF groups (57%, p = 0.0314; 64%, p = 0.0464, respectively). The absolute maximum changes in HDL-c from baseline also showed lower values in the elderly subjects than in the young subjects for the Total/Total, NF/NF, and NF/IF groups (51%, p = 0.0119; 56%, p = 0.0330; 52%, p = 0.0103, respectively).

**Table 3:** Pharmacodynamic parameters of rosuvastatin in young and elderly subjects after oral administration of 20 mg rosuvastatin for 21 days according to OATP1B1/BCRP phenotypes.

OATP1B1/BCRP phenotypes	LDL-c				TC				TG				HDL-c			
	Max change from baseline (%)		AUEC (%·day)		Max change from baseline (%)		AUEC (%·day)		Max change from baseline (%)		AUEC (%·day)		Max change from baseline (%)		AUEC (%·day)	
	Young	Elderly	Young	Elderly	Young	Elderly	Young	Elderly	Young	Elderly	Young	Elderly	Young	Elderly	Young	Elderly
**Total population**																
Total/Total (n = 32) vs (n = 20)	−56.9 ± 7.2	−50.4 ± 15.9	−895 ± 123	−722 ± 262	−33.3 ± 9.5	−33.7 ± 11.3	−511 ± 178	−486 ± 190	−36.8 ± 15.6	−30.7 ± 19.4	−371 ± 273	−259 ± 356	19.3 ± 14.8	9.5 ± 10	210 ± 240	91.4 ± 146
	NS		0.0107		NS		NS		NS		NS		0.0119		0.0314	
**OATP1B1 NF**																
NF/NF (n = 10) vs (n = 7)	−53.5 ± 7	−47.7 ± 8.4	−841 ± 133	−663 ± 158	−30.7 ± 11.4	−33.6 ± 7.8	−469 ± 235	−467 ± 123	−38.8 ± 14.2	−35.6 ± 20.6	−434 ± 261	−377 ± 366	21.5 ± 19.7	9.5 ± 11.4	227 ± 298	105 ± 183
	0.033		0.0063		NS		NS		NS		NS		0.033		NS	
NF/IF (n = 9) vs (n = 8)	−59.5 ± 7.1	−47.6 ± 21.1	−938 ± 102	−708 ± 320	−36.4 ± 6.9	−30.2 ± 14.8	−551 ± 116	−454 ± 240	−37.6 ± 11.2	−21.8 ± 19.4	−373 ± 231	−146 ± 382	17.2 ± 9.1	8.2 ± 9.2	182 ± 161	65.4 ± 140
	NS		NS		NS		NS		0.0464		NS		0.0103		0.0464	
NF/LF (None) vs (n = 1)	—	−74.5	—	—1154	—	−47.4	—	−733	—	−57.9	—	−389	—	2.4	—	25.0
**OATP1B1 IF**																
IF/NF (n = 7) vs (n = 2)	−60.6 ± 4.3	−51.5 ± 2.7	−946 ± 56.6	−640 ± 92.3	−36.0 ± 9.3	−34.1 ± 1.1	−565 ± 157	−430 ± 146	−42.1 ± 10.6	−25.8 ± 9.1	−407 ± 206	−21.5 ± 395	20.9 ± 13.5	23.5 ± 2.1	210 ± 240	228 ± 101
	NS		NS		NS		NS		NS		NS		NS		NS	
IF/IF (n = 4) vs (n = 2)	−56.3 ± 8.4	−57.9 ± 20.8	−883 ± 202	−849 ± 410	−28.8 ± 9.4	−40.3 ± 12.3	−432 ± 207	−611 ± 242	−23.3 ± 31.7	−40.8 ± 8.0	−162 ± 507	−469 ± 44.1	22.9 ± 10.9	4.6 ± 3.0	346 ± 157	42.8 ± 24.3
	NS		NS		NS		NS		NS		NS		NS		NS	
**OATP1B1 LF**																
LF/NF (n = 1) vs (None)	−44.9	—	−753	—	−23.4	—	−406	—	−29.1	—	−246	—	24.2	—	280	—
LF/IF (n = 1) vs (None)	−56.5	—	−868	—	−41.2	—	−628	—	−35.2	—	−422	—	−13.0	—	−316	—
BCRP NF Young vs Elderly	NS		0.0013		NS		NS		NS		NS		NS		NS	

Data are expressed as mean values ± standard deviation.

AUEC, area under the effect-time curve; LDL-c, Low-density lipoprotein cholesterol; TC, Total cholesterol; TG, Triglycerides; HDL-c, High-density lipoprotein cholesterol; NF, normal function; IF, intermediate function; LF, low function; n, number of subjects; NS, not significant.

Based on multiple linear regression analysis, a higher AUEC and maximum change from baseline in LDL-c were significantly associated with only the age group (Supplementary Table S1). The models for AUEC and maximum change from baseline in LDL-c explained 15.5 and 5.8% of the variability, respectively. The reduced function of BCRP and baseline LDL-c were also not significantly associated with higher AUEC or maximum change from baseline in LDL-c, respectively, so these findings were not retained in the final model (p = 0.0657 and 0.1580, respectively). Other independent variables, including reduced function of OATP1B1, sex, body weight or BMI, serum creatinine, and concomitant diseases of hypertension or benign prostatic hyperplasia, were not significantly associated with the dependent variables. The effects of other genetic polymorphisms of *SLCO1B1* on PDs were found to be small (Supplementary Table S3).

### PK-PD relationship of rosuvastatin

In both elderly and young subjects, the relationship between AUC_tau,ss_ and C_max,ss_ vs. AUECs and maximum changes from baseline in LDL-c did not show a clear tendency when classified by OATP1B1/BCRP phenotypes (Supplementary Fig. S1).

## Discussion

In this study, we investigated the influence of age and polymorphisms in *SLCO1B1* and *ABCG2* on the PKs/PDs of rosuvastatin in healthy elderly and young subjects. We found that mean plasma concentrations of rosuvastatin were higher in the elderly subjects than in the young subjects (9.6% in C_max,ss_ and 23% in AUC_tau,ss_, p > 0.05) regardless of transporter phenotypes. In contrast, a previous study reported that the AUC from time 0 to the last measurable concentration was 6% higher in young subjects than in elderly subjects^18^. This previous study might not be comparable because the subjects received a single 40 mg dose of rosuvastatin orally, which was different from our study design. Although not statistically significant, the age-related exposure difference observed in our study is presumably attributable to decreased clearance or increased absorption of rosuvastatin in the elderly population. Furthermore, without the influence of the reduced function of transporters (NF/NF groups), we observed a 41% increase in AUC_tau,ss_ in the elderly subjects compared with that in the young subjects, although the difference was not statistically significant regardless of body weight adjustment (p > 0.05). However, the half-life of rosuvastatin observed within 24 h in the elderly subjects was not significantly different from that in the young subjects (young: 9.1 h vs. elderly: 8.0 h, p > 0.05). In elderly subjects, we also observed relatively slower absorption of rosuvastatin than in young subjects, which could be attributable to reduced gastric acid secretion^23,24^ and gastric emptying^25^.

Overall, LDL-c reductions (AUECs) in response to multiple-dose administration of rosuvastatin were smaller in the elderly subjects than in the young subjects, and the differences were significant. Although it has previously been reported that the efficacy of rosuvastatin is similar in elderly and young patients^19,21^, the results of the present study indicate otherwise. This finding is noteworthy considering that exposure to rosuvastatin and the mean baseline LDL-c level were higher in the elderly subjects. Although the mechanisms of action underlying these observations remain undetermined, it is conceivable that altered concentrations of receptors and hormonal changes result in an alterations in the sensitivity to rosuvastatin, regardless of changes in drug exposure^26–28^. With regard to HDL-c, rosuvastatin has previously been shown to be effective at increasing HDL-c levels^29,30^, and in the present study, we also observed an increasing trend in both elderly and young subjects. In addition, HDL-c increased more in young subjects than in elderly subjects. A rosuvastatin-induced elevation in HDL-c is known to be positively related to the baseline level of TGs and a decrease in TGs and negatively related to the baseline level of HDL-c^31^. Therefore, it is conceivable that the smaller HDL-c increase in the elderly subjects is a reflection of the smaller mean decrease in TGs and the higher mean baseline level of HDL-c.

In the present study, we also evaluated the influence of OATP1B1 and BCRP phenotypes on the PKs/PDs of rosuvastatin. We noted, for the first time, that *ABCG2* 421C > A is related to higher plasma concentrations of rosuvastatin in elderly subjects, which is similar to the findings in the young subjects. Although the large variability observed makes it hard to reach a conclusion, this phenomenon is probably due to the increased bioavailability of rosuvastatin as a consequence of the decreased intestinal efflux of rosuvastatin and a reduction in elimination through the bile^12^. Furthermore, the subjects with the *ABCG2* 421C > A variant did not show a significant decrease in LDL-c levels compared to those with nonvariants, but they showed slight decreasing tendency in LDL-c levels. This slight tendency might be attributed to an elevation in hepatic exposure as well as systemic exposure to rosuvastatin as a consequence of the inhibition of both gastrointestinal absorption and biliary clearance. These observations are roughly comparable with previous studies showing that the *ABCG2* 421C > A variant is associated with an additional reduction in LDL-c levels from baseline^32,33^.

We also observed that the *SLCO1B1* 521T > C variant was not significantly associated with higher exposure (AUC) to rosuvastatin but observed a slight increasing trend in the young subjects, and this trend was less pronounced in the elderly subjects. Additionally, the T_max_ of rosuvastatin was significantly shortened in the elderly subjects with *SLCO1B1* 521T > C compared with the T_max_ of the noncarriers. These phenomena may be ascribed to a decreased uptake of rosuvastatin from blood into hepatocytes, which is presumably associated with age-related effects^34^. Furthermore, we observed no clear association between *SLCO1B1* 521T > C and the LDL-c response to rosuvastatin. However, a previous animal study demonstrated that systemic exposure to rosuvastatin was eightfold higher in Oatp1a/1b-null mice than in wild-type mice, whereas similar concentrations were observed in the liver and bile^35^. Therefore, we speculate that the small or nonexistent lipid-lowering effect of rosuvastatin in subjects with reduced OATP1B1 activity can occur without influencing exposure in the human liver. This finding supports the hypothesis that alterations in OATP1B1 activity can have a marked effect on the plasma concentration of rosuvastatin, with little influence on liver concentrations resulting in a small therapeutic response^33,36^, whereas we could not even observe any significant association between systemic exposure and OATP1B1 activity in this study. Based on the abovementioned findings, genetic polymorphisms of neither *SLCO1B1* nor *ABCG2* significantly affected the LDL-c response to rosuvastatin.

A large interindividual variability in the PKs/PDs of rosuvastatin was observed in this study. Possible reasons accounting for this variability include the influence of genetic polymorphisms, age-related changes, and comorbidities, particularly in elderly subjects (hypertension; n = 7, benign prostatic hyperplasia; n = 6). Elderly individuals are characterized by declines in the function of many regulatory systems in cells and organs, thus resulting in a reduced homeostatic ability that can contribute to high variability^27^. Additionally, a previous study reported that patients with hypertension had increased plasma concentrations of rosuvastatin compared with the concentrations of those without hypertension, but we could not identify any correlation between comorbidities and PKs/PDs in our study^15^. Although it is not clearly known whether this finding is related to the disease itself or to the concomitantly administered anti-hypertensive drugs, it is conceivable that comorbidities may also partially contribute to this high variability.

Widespread clinical use of rosuvastatin is characterized by enhanced efficacy compared with that of other statin drugs at a relatively low dose and limited drug-drug interactions with commonly prescribed drugs^20^. Genetic polymorphisms related to OATP1B1 and BCRP are possible determinants of the efficacy and safety of rosuvastatin, altering lipid-lowering effects and myopathy risk. In the present study, we found that the efficacy of rosuvastatin was lower in the elderly individuals than in the young individuals, while systemic exposure to rosuvastatin was not significantly higher in the elderly subjects. Therefore, even if there is a difference in the PDs between genotypes in the elderly group, the effect may have been less pronounced. However, there might be larger differences in the PKs/PDs of rosuvastatin among elderly patients with different genotypes in clinical settings.

There are several limitations to this study. First, the study included a relatively small number of subjects, thereby reducing the likelihood of obtaining conclusive results. In particular, there were few subjects with OATP1B1 or BCRP LFs, which makes it difficult to determine the clear effects of genetic polymorphisms. Second, the results may also have been influenced by lower drug adherence in the elderly. The young subjects were administered rosuvastatin at each out-patient visit, whereas the evaluation of drug adherence in the elderly subjects relied on a self-medication log and confirmation by phone for 18 days, which might be inaccurate. Third, both studies were not conducted at the same time; however, the research designs were the same, and the clinical analysis was conducted in the same laboratory to obtain consistent results. Last, the different sampling times for blood lipids in each study might cause a bias in estimating the AUEC of lipids, thereby resulting in the difference in lipid-lowering response between young and elderly subjects. However, the maximum change from baseline (%), as another PD parameter less affected by sampling time, showed a similar trend toward a difference between young and elderly subjects, which could complement the drawback. Despite these limitations, however, we were able to identify how age and genetic polymorphisms tend to affect the PKs/PDs of rosuvastatin. This study is also scientifically meaningful in that it examined for the first time the influences of age and genetic polymorphisms in *SLCO1B1* and *ABCG2* in a study population including elderly individuals.

In conclusion, our results revealed that the reduced function of BCRP is associated with exposure to rosuvastatin in both young and elderly subjects. Genetic polymorphisms of neither *SLCO1B1* nor *ABCG2* significantly affected the lipid-lowering response of rosuvastatin. The extent of the response was smaller in elderly subjects as that in young subjects, even though the systemic exposure was not significantly higher in the elderly. We suspect that the influence of genetic polymorphisms on the PDs of rosuvastatin may be less pronounced in the elderly. However, it is conceivable that the effect of genetic polymorphisms on the PKs/PDs of rosuvastatin might be greater in elderly patients in a larger range of clinical settings, which will require further confirmatory studies.

## Methods

### Study population and design

We conducted two separate clinical trials, one of which involved the participation of 20 healthy elderly Korean subjects (age 65–85 years, conducted in 2015–2016) and the other of which involved 34 healthy young Korean subjects (age 20–50 years, conducted in 2010–2011). All participants provided written informed consent. Precise statistical sample size estimation for the hypothesis test was not required due to the exploratory characteristics of both studies. However, using the study of young subjects as a reference, based on the intrasubject variability (~30%) of the rosuvastatin AUC and the ratio of genotypes (wild to variant as 1:1), a sample size of 34 could reveal a difference over 30% in AUC between two genotype groups with 80% power at a significance level of 0.05^14^.

Both studies were designed as open label, one-sequence, and multiple-dose administration studies. Blood samples were drawn for genotyping the day prior to drug administration. All participants received 20 mg of rosuvastatin (Crestor^®^ tablet; AstraZeneca, Seoul, Republic of Korea) once daily for 21 days. For the elderly subjects, first- and final-dose administrations were checked by the investigators in the hospital, and self-administration of the study drugs was carried out from day 2 to day 20, except for day 11 (out-patient visit). Appropriate drug administration was confirmed through telephone calls and a medication diary. Young subjects received rosuvastatin through out-patient visits from day 2 to day 19 and were admitted to the hospital on day 20.

Potential subjects were excluded from the studies if they had (i) moderate renal impairment (creatinine clearance <60 mL/min), (ii) LDL-c levels below the lower limit of the normal range (<55 mg/dL), (iii) participated in other clinical studies and taken any study drugs within 2 months, or (iv) any abnormal findings on physical examination, vital signs, 12-lead electrocardiogram, or clinical laboratory evaluations.

To determine plasma concentrations of rosuvastatin in the steady state, blood samples (6 mL for each) were collected before the final dose on day 21 and 1, 2, 3, 4, 5, 8, 12, and 24 h after dosing. All blood samples were collected in sodium heparin tubes and immediately separated after centrifugation for 10 min. Thereafter, the plasma samples were mixed with 0.2 mol/L (pH 4.0) sodium acetate buffer (plasma: buffer = 3: 1) and stored at -70 °C until analysis. In addition, blood samples (4 mL for each) were collected before the first dose on day 1, on days 11 and 22 for the elderly and before the first dose on day 1 and on days 5, 12, and 22 for the young, to examine any changes in blood lipids caused by rosuvastatin, including LDL-c (the primary PD parameter), total cholesterol (TC), TGs, and HDL-c.

The elderly subject study was approved by the Institutional Review Board (IRB) of Seoul National University Bundang Hospital (IRB No. B-1503–289–002), Seongnam, Gyeonggi-Do, Republic of Korea. Similarly, the young subject study was approved by the IRB of Seoul National University Hospital (IRB No. H-1008-016-326), Jongno-gu, Seoul, Republic of Korea. All procedures were conducted in accordance with the recommendations of the Declaration of Helsinki. Both studies were performed in compliance with Good Clinical Practice and appropriate regulatory requirements established in the Republic of Korea.

### Measurement of rosuvastatin concentrations

Plasma concentrations of rosuvastatin were determined by a validated liquid chromatography-tandem mass spectrometry (LC-MS/MS) method, with rosuvastatin-d6 used as an internal standard. For each determination, 200 μL of sample plasma was mixed thoroughly with 50 μL of rosuvastatin-d6 (20 ng/mL in 1 M acetic acid/methanol = 50/50, v/v) and 1.5 mL of diethyl ether and centrifuged at 10786 × *g* for 10 min. The resulting supernatant was collected and subjected to N_2_ evaporation at 40 °C for 20 min. The residual material was then reconstituted with 100 μL of 1 M acetic acid/methanol = 50/50 (v/v) and injected into the LC-MS/MS system after centrifugation at 10786 × *g* for 5 min. A mobile phase of 0.2% formic acid in distilled water and 100% acetonitrile was used at a flow rate of 0.5 mL/min through a Luna 5 µ C_18_(2) 100 A column (50 × 50 mm; Torrance, CA, USA). The MS/MS system was operated with the electrospray ionizer in positive ionization mode. The precursor-to-product ion reactions monitored for rosuvastatin and rosuvastatin-d6 were *m/z* 482.2 → 258.2 and 488.2 → 264.3, respectively, and the retention times for rosuvastatin and rosuvastatin-d6 were 3.08 and 3.07 min, respectively. The lower limit of quantification (LLOQ) for rosuvastatin was 0.2 ng/mL with linear calibration curves in the concentration range of 0.2 to 80 ng/mL (*r*^*2*^ > 0.99). The intraday accuracy and precision of this analysis were within the ranges of 95.8–99.1% and 1.0–4.2%, and the interday accuracy and precision were within the ranges of 97.4–100.1% and 0.5–1.6%, respectively.

### Genotyping and conversion to phenotypes

Genomic DNA was extracted from 200 μL of peripheral whole blood collected from each participant using a QIAamp DNA Blood Mini Kit (QIAGEN GmbH, Germany). Genotyping was performed using TaqMan Allelic Discrimination Assays in an AB 7500 Real-time PCR System (Applied Biosystems, Foster City, CA, USA). PCR mixtures of 10 µL total volume were prepared, containing 5 μL of 2 × TaqMan Genotyping Master mix, 0.5 μL of 20× Drug Metabolism Genotyping Assay Mix, 3.5 μL of DNase-free water, and 1 μL of genomic DNA. Genotyping for the SNPs SLCO1B1*5 (rs4149056, C_30633906_10), SLCO1B1*1B (rs2306283, C_1901697_20), *SLCO1B1* G-11187A (rs4149015, C_32325356_10), and *ABCG2* 421C > A (rs2231142 and C_15854163_70) was performed using validated TaqMan Genotyping Assays purchased from Applied Biosystems. PCRs were carried out as follows: an initial denaturation at 95 °C for 10 min, followed by 50 cycles of denaturation at 95 °C for 15 s and annealing/extension at 60 °C for 1 min. Allelic discrimination results were determined after amplification by performing an end-point read. Analyses were performed using 7500 Real-Time PCR System software ver. 2.0.6 (Applied Biosystems, Foster City, CA, USA).

Transporter phenotypes were converted by genotypes at rs2231142 for *ABCG2* [C/C, NF; C/A, IF; A/A, LF] based on the reported function of BCRP^12,16,34^ and at rs4149056 for *SLCO1B1* (T/T, NF; T/C, IF; C/C, LF) based on the clinical pharmacogenomic implementation consortium for *SLCO1B1*^37^.

### PK/PD assessments

PK parameters for rosuvastatin were determined by noncompartmental analysis based on the actual sampling times using Phoenix^®^WinNonlin^®^ Software (version 6.4; Certara USA, Inc., Princeton, NJ 08540, USA). The primary PK parameters for this study were as follows: AUC_tau,ss_, calculated using the linear up/log down trapezoidal rule, and C_max,ss_. The secondary PK parameters were T_max_, the apparent clearance at steady state (CL_ss_/F), and the terminal elimination half-life (t_1/2_) derived by ln2/λ_z_, where λ_z_ is the terminal elimination rate constant. After 21 days of treatment, the PD endpoints exhibited the maximum %change in lipids from baseline and %change in the AUEC of lipids from baseline from day 1 to day 22. AUECs were calculated using the trapezoidal rule.

### Statistical analysis

Statistical analyses were performed using SAS version 9.4 (SAS Institute Inc., Cary, NC, USA). To evaluate the effect of the OATP1B1/BCRP phenotype and age on rosuvastatin PKs/PDs, we used parametric and nonparametric analyses: the Kruskal–Wallis test was used for comparison of more than three groups, and the two-sample *t*-test and the Mann-Whitney *U* test were used for two group comparisons. Values of p < 0.05 were considered statistically significant. The PK/PD relationship was evaluated by simple linear regression analysis in elderly and young subjects classified by OATP1B1/BCRP phenotypes.

Multiple linear regression analysis was also performed to evaluate the association of PK and PD with independent variables. Independent variables were genotypic factors by OATP1B1 and BCRP and nongenotypic factors including age or class (elderly for young), sex, body weight or BMI, serum creatinine, baseline levels of lipids, and concomitant medications or diseases of hypertension and benign prostatic hyperplasia. Regression analysis was conducted using a stepwise approach. Each independent variable was evaluated for association with a cut-off p-value (0.20) for inclusion in the model and retained if the p-value was <0.05 in the final multiple linear regression model.

### Principal investigator

The authors confirm that the principal investigators for this paper are Jae-Yong Chung and Kyung-Sang Yu, who had direct clinical responsibility for subjects. Clinical trial registrations at ClinicalTrials.gov: NCT03715101, NCT01218347.

## Supplementary information

Supplementary information

## Data Availability

The datasets generated during and/or analysed during the current study are available from the corresponding author on reasonable request.
