# Supplementary material for: Influence of OATP1B1 and BCRP polymorphisms on the pharmacokinetics and pharmacodynamics of rosuvastatin in elderly and young Korean subjects
Source: Sci Rep. 2019 Dec 19;9:19410. doi: 10.1038/s41598-019-55562-4 (PMC6923423; doi:10.1038/s41598-019-55562-4)
Supplement: Supplementary file 1 — Supplementary information [file 41598_2019_55562_MOESM1_ESM.pdf]

Influence of OATP1B1 and BCRP polymorphisms on the Pharmacokinetics and Pharmacodynamics of Rosuvastatin in elderly and young Korean subjects

Yun Kim, PhD[1], Seonghae Yoon, MD, PhD[2], Yewon Choi, MD, PhD[1], Seo Hyun Yoon, PhD[1], Joo-Youn Cho, PhD[1], In-Jin Jang, MD, PhD[1], Kyung-Sang Yu, MD, PhD[1†], Jae-Yong Chung, MD, PhD[2†*]

[1]Department of Clinical Pharmacology and Therapeutics, Seoul National University College of Medicine and Hospital, Seoul, Korea

[2]Department of Clinical Pharmacology and Therapeutics, Seoul National University College of Medicine and Bundang Hospital, Seongnam, Korea

[†]Principal investigator

The authors confirm that the Principal Investigators for this paper are Jae-Yong Chung and Kyung-Sang Yu that had direct clinical responsibility for subjects

Clinical trials registration in ClinicalTrials.gov: NCT03715101, NCT01218347

***Corresponding author:**

Jae-Young Chung

Associate Professor

Department of Clinical Pharmacology and Therapeutics

Seoul National University College of Medicine and Bundang Hospital

Seongnam, Republic of Korea

Phone: +82-31-787-3955, E-mail: jychung@snubh.org, jychung12@gmail.com

Supplementary Figure S1. Relationship between pharmacokinetics ($C_{max,ss}$ and $AUC_{tau,ss}$) and pharmacodynamics [area under the time-effect curve _low-density lipid (AUEC_LDL), Max %change from baseline (Max %Cfb)_LDL] in (a) young and (b) elderly subjects classified by OATP1B1/BCRP phenotypes. NF, normal function; IF, intermediate function; LF, low function.

(a) Young

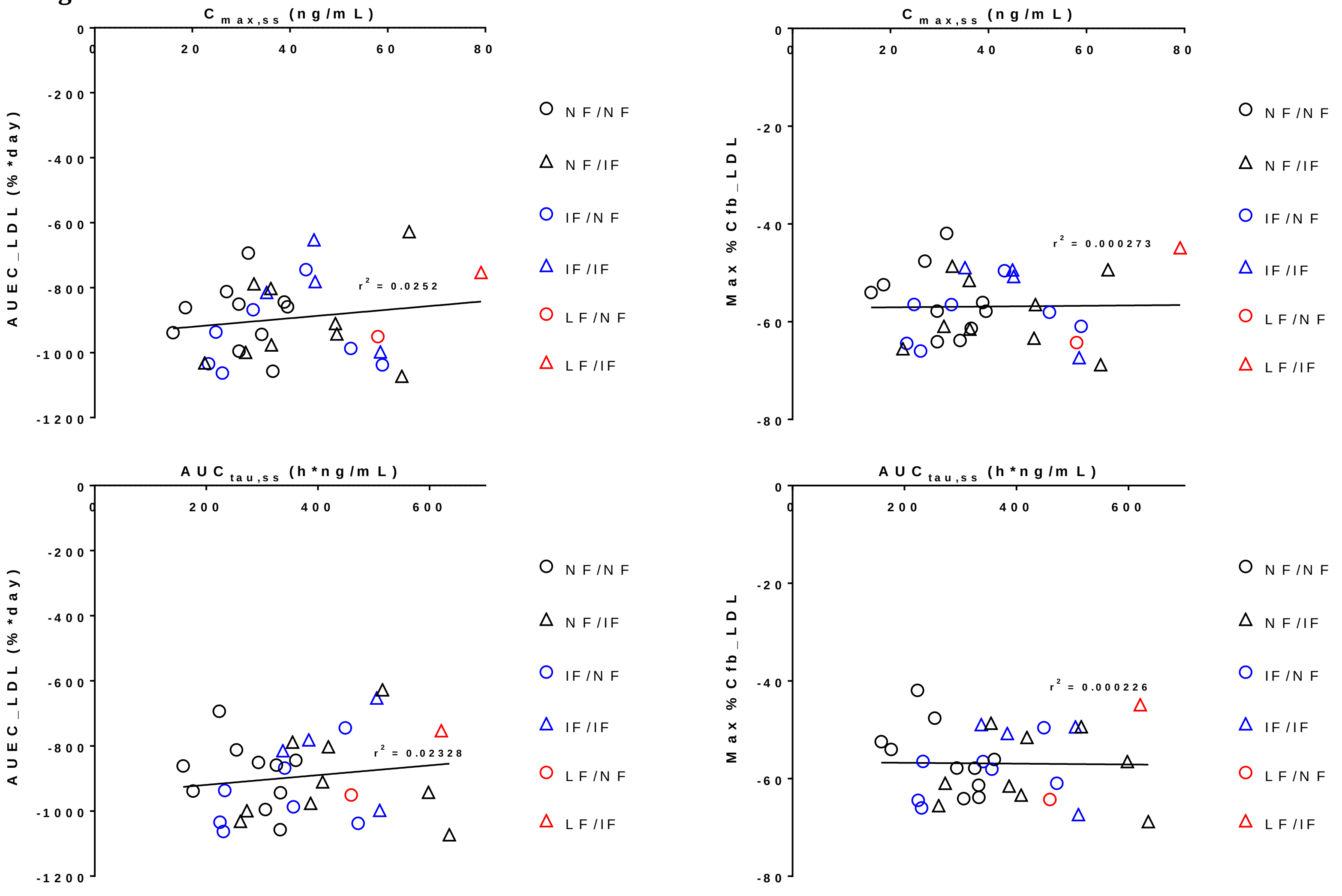

(b) Elderly

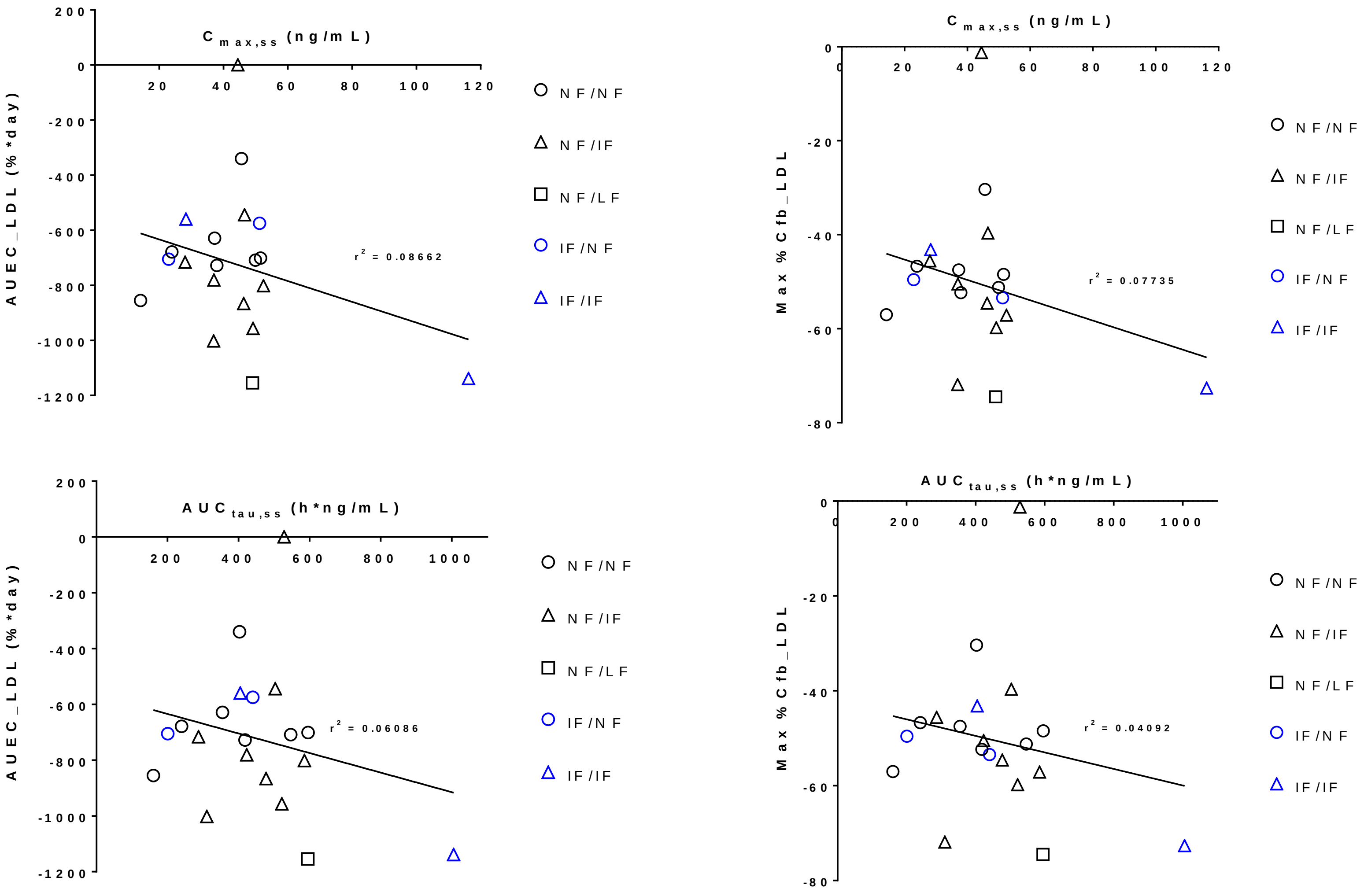

C max,ss (ng/mL)
AUEC_LDL (%*day)
200
0
-200
-400
-600
-800
-1000
-1200
20
40
60
80
100
120
r² = 0.08662
NF/NF
NF/IF
NF/LF
IF/NF
IF/IF
C max,ss (ng/mL)
Max %Cfb_LDL
0
-20
-40
-60
-80
r² = 0.07735
AUC tau,ss (h*ng/mL)
200
400
600
800
1000
r² = 0.06086
AUC tau,ss (h*ng/mL)
r² = 0.04092

Systemic exposure to rosuvastatin showed an almost flat or negative relationship with AUECs and maximum changes from baseline in the young subjects (r^2: 0.000226–0.0252). In contrast, there was a positive relationship between PK and PD parameters in the elderly subjects, although its extent was relatively small (r^2: 0.04092–0.08662). A similar pattern was observed when we evaluated the relationship between PK and the difference in lipids (day 1–day 22, data not shown).

Supplementary Table S1. Analysis of effect of independent variables on the PK/PD parameters of rosuvastatin by multiple linear regression

Variables	$AUC_{tau,ss}$		AUEC in LDL-c		Maximum change from baseline in LDL-c	
	Standardized effect (β)	p-value	Standardized effect (β)	p-value	Standardized effect (β)	p-value
BCRP variants (1=NF, 2=IF, 3=LF)	0.2862	0.0397	-	-	-	-
Class (Young=0, Elderly=1)	-	-	0.4143	0.002	0.2762	0.047

The adjusted R^2 of $AUC_{tau,ss}$, AUEC in LDL-c and maximum change from baseline in LDL-c are 0.064, 0.155 and 0.058, respectively. $AUC_{tau,ss}$, area under the concentration-time curve from 0 to the dosing interval at steady-state; AUEC, area under the effect curve; LDL-c, low-density lipoprotein cholesterol; BCRP, breast cancer resistance protein; NF, normal function; IF, intermediate function; LF, low function

Supplementary Table S2. Pharmacokinetic parameters of rosuvastatin in young and elderly subjects after administration of 20 mg of rosuvastatin for 21 days according to *SLCO1B1* genetic polymorphisms (388A>G, -11187G>A)

Variable		Young vs Elderly					
		***SLCO1B1* 388A>G**		**p-value**	***SLCO1B1* -11187G>A**		**p-value**
		**AA or AG (n=10)**	**GG (n=32) vs. (n=10)**		**GG (n=21) vs. (n=16)**	**GA or AA (n=11) vs. (n=4)**	
$T_{max,ss}$ (h)	Young	-	4.0 [2.0 - 5.0]	-	4.0 [2.0 - 5.0]	4.0 [2.0 - 5.0]	0.367
	Elderly	5.0 [2.0 - 5.0]	5.0 [2.0 - 5.0]	0.684	5.0 [2.0 - 5.0]	4.0 [2.0 - 5.0]	0.437
p-value		-	0.026		0.047	0.851	
$C_{max, ss}$ (µg/L)	Young	-	39.6 ± 14.9 (37.5)	-	36.6 ± 13.6 (37.2)	45.5 ± 16.1 (35.4)	0.168
	Elderly	54.6 ± 22.1 (40.5)	32.3 ± 10.7 (33.1)	0.001	42.1 ± 10.6 (25.3)	48.9 ± 45.0 (92.0)	0.335
p-value		-	0.236		0.068	0.412	
$AUC_{tau,ss}$ (µg*h/L)	Young	-	366.8 ± 125.6 (34.2)	-	348.1 ± 127.1 (36.5)	402.4 ± 120.2 (29.9)	0.194
	Elderly	538.9 ± 185.2 (34.4)	361.1 ± 140.6 (38.9)	0.029	443.9 ± 127.8 (28.8)	474.4 ± 363.6 (76.6)	0.494
p-value		-	0.988		0.023	0.753	
$t_{1/2}$ (h)	Young	-	9.1 ± 2.5 (27.0)	-	9.5 ± 2.5 (26.7)	8.4 ± 2.3 (27.0)	0.254
	Elderly	7.1 ± 0.9 (12.6)	8.8 ± 2.7 (30.6)	0.052	8.0 ± 2.4 (30.0)	8.0 ± 0.6 (7.3)	0.437
p-value		-	0.531		0.018	0.753	
CL_{ss}/F (L/h)	Young	-	61.5 ± 22.8 (37.0)	-	65.2 ± 24.4 (37.5)	54.4 ± 18.0 (33.2)	0.194
	Elderly	40.4 ± 11.6 (28.8)	65.0 ± 29.4 (45.3)	0.029	50.9 ± 23.7 (46.5)	59.6 ± 33.6 (56.3)	0.494
p-value		-	0.988		0.023	0.753	

Data are expressed as mean values ± SD (CV %) except for $T_{max,ss}$ as median [range]. $C_{max,ss}$, maximum observed concentration at steady state; $AUC_{tau,ss}$, area under the concentration-time curve from 0 to the dosing interval of 24 hours for rosuvastatin; CL_{ss}/F, oral clearance at steady state; $t_{1/2}$, half life; $T_{max,ss}$, time to maximum plasma concentration at steady state. Comparison was performed each by Mann-Whitney U test.

Supplementary Table S3. Pharmacodynamic parameters of rosuvastatin in young and elderly subjects after oral administration of 20 mg of rosuvastatin for 21 days according to *SLCO1B1* genetic polymorphisms (388A>G, -11187G>A)

Lipid		Parameters	Young vs Elderly					
			SLCO1B1 388A>G		p-value	*SLCO1B1* -11187G>A		p-value
			AA or AG (n=10)	GG (n=32) vs. (n=10)		GG (n=21) vs. (n=16)	GA or AA (n=11) vs. (n=4)	
LDL-c	Young	Max change from baseline (%)	-	-56.9 ± 7.2	-	-57.6 ± 7	-55.7 ± 7.7	0.484
	Elderly		-53.9 ± 13	-46.8 ± 18.3	0.247	-49.8 ± 16.7	-52.8 ± 13.5	0.682
		p-value	-	0.019		0.04	0.489	
	Young	AUEC (%·day)	-	-894.7 ± 123.2	-	-908.6 ± 121	-868.2 ± 128.7	0.387
	Elderly		-763.4 ± 239.8	-680.6 ± 288.8	0.494	-707.5 ± 270.6	-780 ± 249.7	0.633
		p-value	-	0.002		0.004	0.28	
TC	Young	Max change from baseline (%)	-	-33.3 ± 9.5	-	-33.7 ± 9.2	-32.5 ± 10.3	0.734
	Elderly		-34.8 ± 9.8	-32.5 ± 13	0.796	-32.8 ± 12	-37.2 ± 8	0.892
		p-value	-	0.873		0.964	0.412	
	Young	AUEC (%·day)	-	-511.3 ± 177.8	-	-526.9 ± 185.3	-481.7 ± 166.9	0.327
	Elderly		-495.4 ± 184.1	-476.3 ± 204.9	0.971	-464.3 ± 197.2	-571.9 ± 146.7	0.323
		p-value	-	0.328		0.108	0.412	

TG	Young	Max change from baseline (%)	-	-36.8 ± 15.6	-	-40.5 ± 17.2	-29.9 ± 8.8	0.012
	Elderly		-32.3 ± 16.9	-29.2 ± 22.4	0.734	-31.2 ± 20	-28.7 ± 19.5	0.822
		p-value	-	0.231		0.108	0.753	
	Young	AUEC (%·day)	-	-370.5 ± 272.7	-	-395.3 ± 312.9	-323 ± 175.8	0.485
	Elderly		-310.3 ± 318.6	-207.4 ± 399.2	0.532	-294.6 ± 346.2	-115.9 ± 408.4	0.383
		p-value	-	0.149		0.36	0.661	
HDL-c	Young	Max change from baseline (%)	-	19.3 ± 14.8	-	22.8 ± 14.3	12.7 ± 14	0.067
	Elderly		8.9 ± 10.6	10.2 ± 9.9	0.779	9.5 ± 10.5	9.7 ± 8.5	0.892
		p-value	-	0.076		0.002	0.753	
	Young	AUEC (%·day)	-	209.9 ± 240.4	-	265.3 ± 240.4	104.3 ± 211.9	0.071
	Elderly		64.1 ± 145.6	118.6 ± 148.2	0.417	92 ± 161.9	88.7 ± 57.8	0.969
		p-value	-	0.265		0.018	0.851	

Data presented as mean ± standard deviation. AUEC, area under the effect-time curve; LDL-c, Low-density lipoprotein cholesterol; TC, Total cholesterol; TG, Triglycerides; HDL-c, High-density lipoprotein cholesterol; NF, normal function; IF, intermediate function; LF, low function
Comparison was performed each by Two sample t-test or Mann-Whitney U test.
